# Robotic-assisted laparoscopic inguinal hernia repair after previous transabdominal prostatectomy

**DOI:** 10.1007/s00464-021-08497-9

**Published:** 2021-04-01

**Authors:** M. Dewulf, L. Aspeslagh, F. Nachtergaele, P. Pletinckx, F. Muysoms

**Affiliations:** 1 Department of Surgery, Maria Middelares, Ghent, Belgium; 2 Department of Surgery, Maastricht University Medical Center+, Maastricht, The Netherlands; 3 Department of Surgery, Maria Middelares, Buitenring Sint-Denijs 30, 9000 Ghent, Belgium

**Keywords:** Inguinal hernia, Groin hernia, Robotic-assisted surgery, Prostatectomy

## Abstract

**Background:**

Transabdominal prostatectomy results in scarring of the retropubic space and this might complicate subsequent preperitoneal dissection and mesh placement during minimally invasive inguinal hernia repair. Therefore, it suggested that an open anterior technique should be used rather than a minimally invasive posterior technique in these patients.

**Methods:**

In this single-center study, a retrospective analysis of a prospectively maintained database was performed. All patients undergoing inguinal hernia repair after previous transabdominal prostatectomy were included in this analysis, and the feasibility, safety, and short-term outcomes of open and robotic-assisted laparoscopic inguinal hernia repair were compared.

**Results:**

From 907 inguinal hernia operations performed between March 2015 and March 2020, 45 patients met the inclusion criteria. As the number of patients treated with conventional laparoscopy was very low (*n* = 2), their data were excluded from statistical analysis. An open anterior repair with mesh (Lichtenstein) was performed in 21 patients and a robotic-assisted laparoscopic posterior transabdominal repair (rTAPP) in 22. Patient characteristics between groups were comparable. A transurethral urinary catheter was placed during surgery in 17 patients, most often in the laparoscopic cases (15/22, 68.2%). In the rTAPP group, a higher proportion of patients was treated for a bilateral inguinal hernia (50%, vs 19% in the Lichtenstein group). There were no intraoperative complications and no conversions from laparoscopy to open surgery. No statistically significant differences between both groups were observed in the outcome parameters. At 4 weeks follow-up, more patients who underwent rTAPP had an asymptomatic seroma (22.7% vs 5% in the Lichtenstein group) and two patients were treated postoperatively for a urinary tract infection (4.7%).

**Conclusion:**

A robotic-assisted laparoscopic approach to inguinal hernia after previous transabdominal prostatectomy seems safe and feasible and might offer specific advantages in the treatment of bilateral inguinal hernia repairs.

With an estimated 1.1 million diagnoses worldwide in 2012, prostate cancer is one of the most common cancers in men, accounting for 15% of all cancers diagnosed in the male population. Partly due to the widespread use of prostate-specific antigen (PSA) screening, its incidence is still on the rise [1, 2]. Surgery remains the cornerstone in its treatment and can be performed by open retropubic radical prostatectomy (RRP), laparoscopic radical prostatectomy (LRP), or robotic-assisted laparoscopic prostatectomy (RALP). Regardless of the technique used, the surgical treatment of prostate cancer traditionally involves an extensive dissection of the retropubic space. This results in scarring of the Retzius space, which complicates subsequent minimally invasive posterior inguinal hernia repair (IHR) in the preperitoneal plane [1, 3, 4]. For this reason, current guidelines advocate an open anterior inguinal hernia repair in these patients [4]

Transabdominal surgery for prostate cancer has been identified as an independent risk factor for the development of an inguinal hernia, with an estimated incidence of 15.9% after RRP, and 6.7% after LRP [2, 5, 6]. A nation-wide Swedish population study in 28,608 patients observed an almost fourfold increase in inguinal hernia repair after radical prostatectomy [7]. The exact mechanism remains under debate and probably is multifactorial [8, 9]. Recent meta-analysis identified increasing age, low body mass index (BMI), presence of a subclinical inguinal hernia, previous hernia repair, and an anastomotic stricture as risk factors for the development of an inguinal hernia after radical prostatectomy [5].

In the treatment of a primary inguinal hernia, a minimally invasive posterior repair is now suggested as the gold standard, provided a surgeon with specific expertise is available. Both a transabdominal preperitoneal (TAPP) and a totally extraperitoneal (TEP) repair involve a dissection of the preperitoneal and retropubic space and result in a lower incidence of postoperative and chronic pain, when compared to open surgery [1, 4]. Furthermore, these techniques offer specific advantages in the treatment of bilateral inguinal hernias, and current guidelines strongly recommend their use in case of bilateral disease [4]. Extensive experience in minimally invasive inguinal hernia repair, along with the introduction of robotic surgery, has led to a dramatic increase in the indications for minimally invasive abdominal wall and inguinal hernia surgery. However, evidence on laparoscopic inguinal hernia repair after previous transabdominal prostatectomy is still lacking, and only 5 patient series on the topic have been published [1, 3, 10–13].

## Objectives

This study aims to investigate the safety, feasibility, and short-term outcomes of a minimally invasive posterior approach in patients after previous transabdominal prostatectomy. In this retrospective analysis of a prospectively maintained database, the intraoperative characteristics and short-term outcomes of laparoscopic IHR (both conventional and robotic-assisted) are examined and compared with open surgery in these patients.

## Methods

### Setting

This study was conducted at the surgical department of Maria Middelares Hospital (Ghent, Belgium). In a single-center observational case–control design, data of a prospectively maintained database were retrospectively analyzed. Included patients were treated between March 2015 and March 2020. Surgery was performed by one surgeon with extensive experience in both open and minimally invasive IHR. The study protocol was approved by the local ethics committee on October 7th, 2020, before the start of inclusions, with reference number MMS.2020.067. All patients and surgical data were prospectively entered in the EuraHS (European registry for abdominal wall hernias) database at the time of surgery and at the 4 weeks follow-up visit [14]. For analysis, data were extracted in an anonymized manner. Before closure of the database, data and missing values were double checked.

### Patients

All patients with a history of transabdominal prostatectomy scheduled to undergo uni- or bilateral IHR during the period March 1st, 2015–March 31st, 2020, were eligible for inclusion. Exclusion criteria were as follows: age under 18, inguinal hernia repair without mesh placement, and open inguinal hernia repair with a technique other than Lichtenstein. All patients were scheduled for a standard clinical outpatient follow-up visit with the surgeon at 4 weeks postoperatively.

### Surgical technique

All operations were performed under general anesthesia. A single prophylactic dose of 2 g cefazoline (Cefacidal, Bristol-Myers Squibb, Braine-l’Alleud, Belgium) was administered in case of open surgery, and no prophylactic antibiotics were given in case of minimally invasive IHR. Patients were instructed to void prior to surgery, and a transurethral urinary catheter was placed during surgery in 17 patients, most often in the laparoscopic cases (15/22, 68.2%), according to the surgeon’s preference. Hernia repair was performed according to the standard surgical principles, and mesh placement occurred after achieving the critical view of the myopectineal orifice (MPO) in posterior repairs [15].

#### Open surgery

In open surgery, an iodine-impregnated drape was used to cover the surgical field. Surgery was performed using a 6-cm long incision, and a standard Lichtenstein technique was used. A self-gripping monofilament polyester mesh (Parietex Progrip™ Self-Fixating Mesh, Medtronic, Minneapolis, MN, US) of 15 by 15 cm was tailored to a mesh with a slit for the cord and a width of 14 cm and a length of 9 cm. No additional sutures were used for fixation of the mesh.

#### Robotic-assisted laparoscopic IHR

Robotic operations were performed using the daVinci Xi system (Intuitive, Sunnyvale, CA, US) with a 0° scope. Three robotic 8 mm trocars were placed on a horizontal line at the umbilicus and on both sides with 7 cm between trocars. Blind entry of the blunt first trocar at the umbilicus was performed to create the pneumoperitoneum at 12 mmHg. Self-gripping monofilament polyester mesh (Parietex Progrip™ Self-Fixating Mesh, Medtronic, Minneapolis, MN, US) was used, with a width of 16 cm and a length of 12 cm for unilateral hernias, and with a width of 28 cm and a length of 13 cm for bilateral hernias. Care was taken to properly close the peritoneum after mesh placement using a barbed suture (V-Loc™ 90, Medtronic, Minneapolis, MN, US). Three robotic instruments were used (monopolar hot shears curved scissors, fenestrated bipolar forceps, and a large needle driver).

### Endpoints and variables

The rate of intrahospital complications (according to the Clavien-Dindo classification) was defined as the primary endpoint [16]. Postoperative complications within 4 weeks after surgery (stratified as none, readmission, seroma and urinary tract infection) were defined as the secondary endpoint. Furthermore, data on duration of surgery, intraoperative complications, intraoperative urinary catheterization, postoperative urinary retention, and postoperative hospital stay (stratified as ambulatory surgery, 1 night or 2 nights postoperative stay) were collected and analyzed. For classification of inguinal hernias, the European Hernia Society classification was used [17].

### Statistical analysis

For descriptive data on patient demographics and outcomes, mean and median values or proportions (n/N) were calculated. Data were checked for distribution and normality using the Kolmogorov–Smirnov and Shapiro–Wilk tests. *P* values were calculated using the Mann–Whitney *U* test or the independent samples *T* test for continuous variables and the Fisher’s exact test for categorical variables. *P* values ≤ 0.05 were considered indicating statistical significance. Data analysis was carried out using Microsoft Excel (Redmond, WE, US) and SPSS Statistics (Northcastle, NY, US). As the number of patients treated with conventional laparoscopy was very low (*n* = 2), their data were excluded from statistical analysis.

## Results

### Patient characteristics

**Fig. 1:**
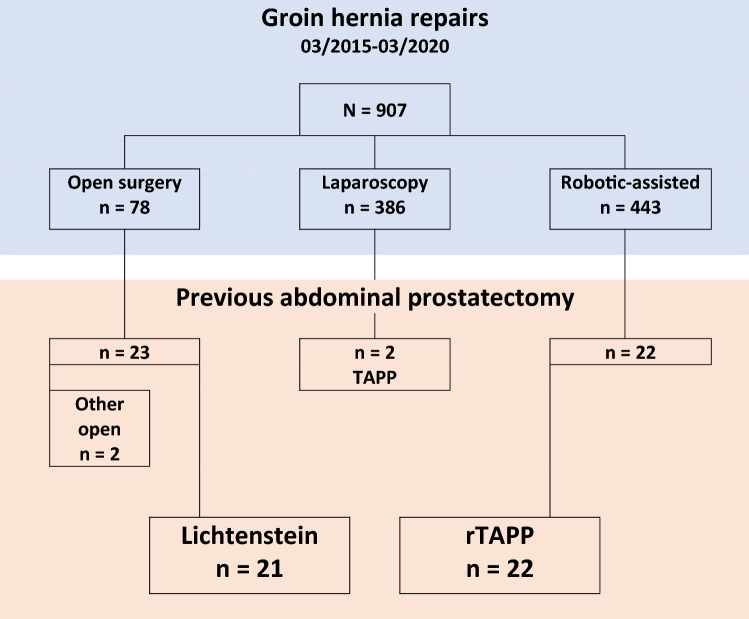
Flow chart for patients included in the analysis. *TAPP* laparoscopic transabdominal preperitoneal inguinal hernia repair, *rTAPP* robotic-assisted transabdominal preperitoneal inguinal hernia repair

**Fig. 2:**
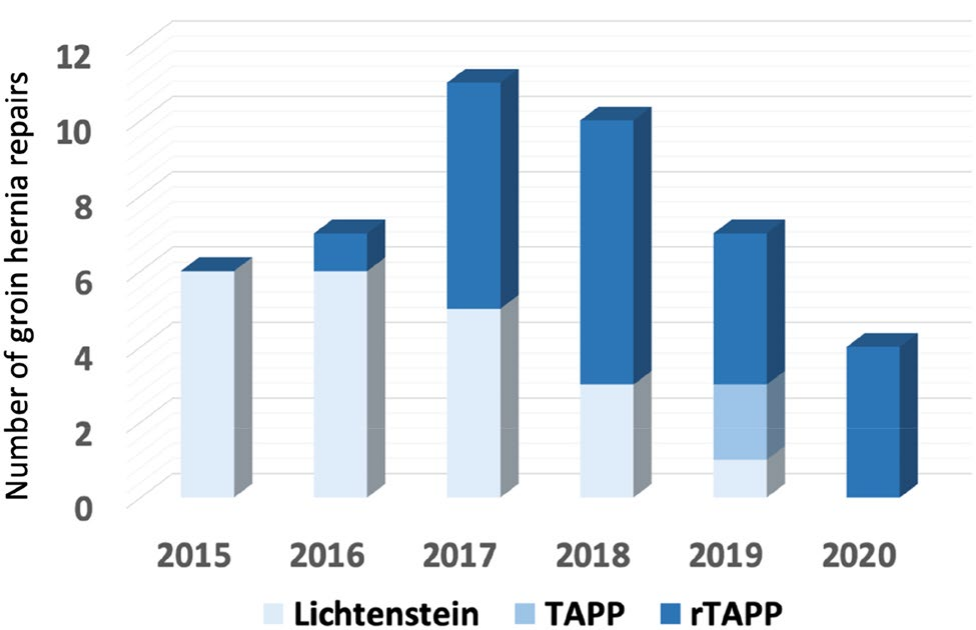
Evolution in inguinal hernia repair technique after prostatectomy

During the study period, 907 patients underwent IHR at our center. Among them, 47 patients had a history of transabdominal prostatectomy. Eventually, 43 patients met the inclusion criteria and were included for further analysis. Of the included patients, 21 were treated by open surgery, and 22 patients underwent minimally invasive IHR. A flowchart of patient numbers is depicted in Fig. 1. The evolution in the technique used over time is shown in Fig. 2. With the introduction of the robotic platform to our practice in September 2016, a clear evolution can be seen from open surgery towards robotic-assisted laparoscopic surgery.

### Outcomes

Patient characteristics and surgical data are listed in Table 1. When comparing the Lichtenstein group with the group that underwent robotic-assisted TAPP (rTAPP), baseline characteristics were similar. Regarding age, years since prostatectomy, prostatectomy technique, comorbidities, and BMI, no statistically significant differences were observed. In the rTAPP group, a higher proportion of patients was treated for a bilateral inguinal hernia (50%, vs 19% in the Lichtenstein group). Three patients in the rTAPP group and two patients in the Lichtenstein group underwent previous IHR. Three patients with a femoral hernia after previous prostatectomy were all treated by minimally invasive approach. One patient underwent emergency surgery and was treated with open surgery.

**Table 1:** Baseline characteristics

	Lichtenstein (*N* = 21)	rTAPP (*N* = 22)	*P* value
Age at time of surgery (years)	73.6 (72.0)	73.8 (75.4)	0.304^a^
Years since prostatectomy	5.6 (4.0)	7.7 (7.5)	0.212^b^
Prostatectomy approach			1.000^c^
Open prostatectomy	57.1% (12/21)	54.5% (12/22)	
Robotic-assisted prostatectomy	42.9% (9/21)	45.5% (10/22)	
Hernia side			
Bilateral	19.0% (4/21)	50.0% (11/22)	0.055^c^
Left side	47.6% (10/21)	27.3% (6/22)	0.215^c^
Right side	33.3% (7/21)	22.7% (5/22)	0.510^c^
EHS hernia classification			
Hernia size			
1	14.3% (3/21)	–	0.108^c^
2	57.1% (12/21)	68.2% (15/22)	0.537^c^
3	28.6% (6/21)	31.8% (7/22)	1.000^c^
Hernia location			
Medial	33.3% (7/21)	45.5% (10/22)	0.537^c^
Lateral	81.0% (17/21)	81.8% (18/22)	1.000^c^
Femoral	–	13.6% (3/22)	0.233^c^
Recurrent hernia	9.5% (2/21)	13.6% (3/22)	1.000^c^
Emergency surgery	4.8% (1/21)	–	0.488^c^
Comorbidities			
Anticoagulation	52.4% (11/21)	36.4% (8/22)	0.364^c^
Previous hernia surgery	33.3% (7/21)	27.3% (6/22)	0.747^c^
Smoker	–	13.6% (3/22)	0.233^c^
Body mass index (kg/m^2^)			
< 25	57.1% (12/21)	63.6% (14/22)	0.760^c^
25–30	28.6% (6/21)	36.4% (8/22)	0.747^c^
≥ 30	14.3% (3/21)	–	0.108^c^

Data are % (n/N) or mean (median)

*rTAPP* robotic-assisted transabdominal preperitoneal inguinal hernia repair, *EHS* European hernia society

^a^Difference between the two groups according to independent samples *T* test

^b^Difference between the two groups according to Mann–Whitney *U* Test

^c^Difference between the two groups according to Fisher's Exact Test

Outcome data are listed in Table 2. Regarding intraoperative characteristics, both overall operative times and duration of surgery in unilateral hernias were significantly longer in the rTAPP group. This difference was no longer statistically significant in case of bilateral repair. There were no intraoperative complications and no conversions from laparoscopy to open surgery. A transurethral urinary catheter was placed during surgery in 17 patients, most often in the laparoscopic cases (15/22, 68.2%).

**Table 2:** Clinical outcome data

	Lichtenstein (*N* = 21)	rTAPP (*N* = 22)	*P* value^a^
Duration of surgery (min)			
Overall	43.2 (41.0)	78.7 (65.5)	< 0.0001
Unilateral hernia	39.6 (35.0)	69.5 (64.0)	< 0.0001
Bilateral hernias	58.5 (55.0)	87.8 (83.0)	0.078
Intraoperative complications	–	–	
Perioperative urinary catheter	9.5% (2)	68.2% (15)	< 0.0001
Intrahospital complications^b^			
None	100% (21)	95.5% (21)	1.000
Grade I–IIIa	–	4.5% (1)	
Grade IIIb–V	–	–	
Postoperative urinary retention	–	4.5% (1)	1.000
Postoperative hospital stay			
Ambulatory surgery	14.3% (3)	27.3% (6)	0.457
1 night	66.7% (14)	59.1% (13)	0.755
≥ 2 nights	19.0% (4)	13.6% (3)	0.698
Postoperative complications^c^			
None	85.0% (17)	72.7% (16)	0.460
Readmission	5.0% (1)^d^	–	0.476
Seroma	5.0% (1)	22.7% (5)	0.187
Urinary tract infection	5.0% (1)	4.5% (1)	1.000
Cases lost to follow-up	4.8% (1)	–	0.488

Data are % (n/N) or mean (median)

*rTAPP* robotic-assisted transabdominal preperitoneal inguinal hernia repair

^a^Difference between the two groups according to Fisher's exact test or Mann–Whitney *U* test

^b^According to the Clavien-Dindo classification

^c^During a follow-up period of 4 weeks

^d^Reason for readmission: diverticular bleeding

No statistically significant differences between both groups were observed in the outcome parameters. Mean hospital stay in days was 1.1 (SD 0.7) for the open group and 1.0 (SD 0.8) for the laparoscopic group. Eighty-six percent of the patients were treated ambulatory or with one night stay. Urinary retention requiring catheterization in one patient was the only complication noted during hospitalization. One patient from the Lichtenstein group was readmitted due to diverticular bleeding.

At 4 weeks follow-up, more patients who underwent rTAPP had an asymptomatic seroma (22.7% vs 5.0% in the Lichtenstein group) and two patients were treated postoperatively for a urinary tract infection (4.7%).

## Discussion

### Main results

No intraoperative complications or conversions were observed in our study. Operative times were significantly longer in the group treated with minimally invasive surgery when compared to open surgery. This observation seems to fade in case of bilateral hernia repair. Not surprisingly, these operative times are also considerably longer than duration of surgery by rTAPP in primary IHR in our center, even at the beginning of an observed learning curve [18].

In this patient series, overall postoperative outcomes in open and robotic-assisted laparoscopic IHR after transabdominal prostatectomy are comparable. Although not statistically significant, there was a higher rate of seroma formation in the rTAPP group 4 weeks postoperatively (22.7%). This percentage of seroma formation is slightly higher when compared to IHR by rTAPP at our center in primary inguinal hernias (15%) [19]. We do not routinely plicate the hernia sac in minimally invasive surgery to reduce this ‘dead space’. On the contrary, we do have a habit of resection and ligation of the hernia sac during open surgery, which could partly explain this difference in seroma formation. These findings suggest that a robotic-assisted IHR in these patients is safe and feasible.

### Interpretation

This is the first study to compare minimally invasive surgery to open surgery in IHR after transabdominal prostatectomy. To date, there are only three prospective and two retrospective patient series available on the topic [1].Three of them use a control group of patients who did not have prostate surgery [3, 11, 12] and 2 of them have an uncontrolled design [10, 13]. Besides design, there is large heterogeneity among them regarding sample size, applied technique and prostatectomy approach. The largest currently available study was published by the group of Reinhard Bittner in 2009 and reported on favorable results of TAPP after radical prostatectomy in 214 patients [3]. In the study of Sakon et al., no dissection of the retropubic space was performed and only patients with indirect hernias were included [13]. By avoiding this medial dissection, no critical view of the MPO was obtained before mesh placement, which highly complicates interpretation of their results and limits extrapolation to patients with direct hernias [13, 19]. Generally, our observations are consistent with currently available literature.

Because of comparable outcomes between bilateral and unilateral IHR in laparoscopic surgery, and the possibility to perform a bilateral repair without the need for additional incisions, current guidelines strongly recommend minimally invasive surgery in case of bilateral primary inguinal hernias [4, 20]. In our study, more patients with bilateral disease were treated by rTAPP, and although still longer, the difference in operative times was no longer statistically significant in patients who underwent bilateral IHR. In 4 patients who were preoperatively diagnosed with a unilateral inguinal hernia, the intraoperative diagnosis of a bilateral inguinal hernia was made and a bilateral repair was performed. This partially explains the higher rate of bilateral repairs in the rTAPP group, and highlights another advantage of the minimally invasive transabdominal approach. Furthermore, we believe that there is benefit in visualization and prelevation of lymph nodes along the iliac vessels during minimally invasive inguinal hernia repair in this patient group. Most patients underwent prostatectomy for oncological reasons, and despite good follow-up and staging before the surgical treatment of an inguinal hernia, often enlarged lymph nodes are encountered during surgery. We have a habit of sending them for pathological examination, which could add information on their oncological situation. These observations, along with comparable complication rates between rTAPP and Lichtenstein, advocate the use of minimally invasive surgery, especially in patients with bilateral inguinal hernias or when there is doubt about the diagnosis of a contralateral inguinal hernia.

Regarding the increased incidence of inguinal hernia after prostatectomy, it is generally assumed that the exposure of the retropubic space results in damaging transversalis fascia, the posterior layer of the rectus sheath, and the endopelvic fascia, thereby disrupting the integrity of the posterior wall of the inguinal canal. Furthermore, stretching of Hesselbach’s ligament contributes to a decrease in strength of the internal ring [2, 8, 9]. Minimally invasive and Retzius-sparing techniques seem to decrease the risk of an inguinal hernia by minimizing damage to the region of the myopectineal orifice (MPO) [6, 9, 21]. Several prophylactic measures have been proposed to minimize the risk of inguinal hernia after transabdominal prostatectomy, including a ligation and transection of the processus vaginalis, blunt dissection of the peritoneum close to the internal ring with an isolation of the spermatic cord, or the placement of additional stitches to close the internal ring [6, 22–24]. Finley et al. reported on a concomitant repair of an inguinal hernia during RALP using prosthetic mesh in 36 patients [25], whereas Lee et al. proposed a technique using plugs of hemostatic agents to repair incidentally found inguinal hernias during prostatectomy [26]. Given the high incidence of inguinal hernia after prostatectomy, there is a need for further research on this topic to confirm effectiveness of available techniques. Thereby, continued awareness of this specific problem among urologists is needed to further minimize these numbers.

### Limitations

Besides the retrospective design, this study has several limitations. First, the choice of the surgical technique was not randomized and highly dependent on the preference of the surgeon. One could presume that patient characteristics and BMI influenced the choice of the surgical technique, although BMI and comorbidities were comparable between groups.

Second, the length of follow-up in our study is limited to 4 weeks, as this comprises the standard follow-up in our center after IHR. One of the main advantages of minimally invasive IHR over open surgery is a reduction in postoperative and chronic pain [1, 4]. Due to the retrospective design of this study and the subsequent short follow-up, no data on the topic are available.

Third, minimally invasive surgery in this study was performed robotic-assisted. Currently, rTAPP is not a widespread practice in Europe, mainly due to cost-effectiveness and logistic issues. As observed in Fig. 2, it is the availability of the robotic platform that caused a shift in our practice from open to laparoscopic surgery. This implicates the introduction of the robot to our practice during the inclusion period. Obviously, this also implicates a learning curve during the inclusion period of this study, although no intraoperative complications or conversions were observed. Whether our observations in robotic surgery, performed by surgeons with extensive experience in abdominal wall and robotic-assisted surgery, can be extrapolated to conventional laparoscopy is unclear.

### Indications for future research

As mentioned above, evidence for minimally invasive IHR after transabdominal prostatectomy is scarce and of limited quality, and current guidelines still advocate open surgery in these patients. This stresses the need for prospective studies with a randomization for the surgical technique. Furthermore, outcome parameters indicating quality of life during a longer follow-up period are paramount to conclusively show an advantage of minimally invasive surgery. Along with the need for future studies on rTAPP in these patients, further evidence on conventional laparoscopic techniques is highly warranted.

## Conclusion

A robotic-assisted laparoscopic approach to inguinal hernia after previous transabdominal prostatectomy seems safe and feasible and might offer specific advantages in the treatment of bilateral inguinal hernia repairs.
